# Higher mortality risk among injured individuals in a population-based matched cohort study

**DOI:** 10.1186/s12889-017-4087-0

**Published:** 2017-02-02

**Authors:** Rebecca J. Mitchell, Cate M. Cameron, Rod McClure

**Affiliations:** 1 Australian Institute of Health Innovation, Macquarie University, Level 6, 75 Talavera Road, North Ryde, NSW 2109 Australia; 2 Menzies Health Institute Queensland, Griffith University, Gold Coast, Australia; 3 Harvard Injury Control Research Center, Harvard School of Public Health, Harvard University, Boston, USA

**Keywords:** Injury, Mortality, Comorbidity, Hospitalisation, Survival

## Abstract

**Background:**

Improved understanding of long-term mortality attributable to injury is needed to accurately inform injury burden studies. This study aims to quantify and describe mortality attributable to injury 12 months after an injury-related hospitalisation in Australia.

**Method:**

A population-based matched cohort study using linked hospital and mortality data from three Australian states during 2008–2010 was conducted. The injured cohort included individuals ≥18 years who had an injury-related hospital admission in 2009. A comparison cohort of non-injured people was obtain by randomly selecting from the electoral roll. This comparison group was matched 1:1 on age, gender and postcode of residence. Pre-index injury health service use and 12-month mortality were examined. Adjusted mortality rate ratios (MRR) and attributable risk were calculated. Cox proportional hazard regression was used to examine the effect of risk factors on survival.

**Results:**

Injured individuals were almost 3 times more likely to die within 12 months following an injury (MRR 2.90; 95% CI: 2.76–3.04). Individuals with a traumatic brain injury (MRR 7.58; 95% CI: 5.92–9.70) or injury to internal organs (MRR 7.38; 95% CI: 5.90–9.22) were 7 times more likely to die than the non-injured group. Injury was likely to be a contributory factor in 92% of mortality within 30 days and 66% of mortality at 12 months following the index injury hospital admission. Adjusted mortality rate ratios varied by type of cause-specific death, with MRR highest for injury-related deaths.

**Conclusions:**

There are likely chronic consequences of sustaining a traumatic injury. Longer follow-up post-discharge is needed to consider deaths likely to be attributable to the injury. Better enumeration of long-term injury-related mortality will have the potential to improve estimates of injury burden.

## Background

Measuring the burden of injury accurately is reliant upon good quality information on injury mortality and morbidity at a population level [1]. Global burden of disease (GBD) studies commonly use Disability Adjusted Life Years (DALYs) as a summary measure of population health as it provides information on mortality, morbidity and related disability [2] and can be used to estimate and compare population health burden relative to different health conditions.

DALYs are made up of Years of Life Lost (YLL) to premature mortality and Years of Life Lost to Disability (YLD). Counts of injury-related mortality are used to estimate YLL, which would appear to be a straightforward measure – either through identifying in-hospital mortality in hospital separation data or identifying injury-related deaths using underlying and/or antecedent causes of death in death registration data. However, if the injury occurs months preceding mortality, injury is not always recognised as a contributory cause of death, for example an older person is injured and hospitalised following a fall, and then later dies from a complication, such as pneumonia. As there can be a time delay for deaths following injury, these deaths are often attributed to other underlying conditions [1, 2], particularly for older individuals and individuals who experience comorbid conditions [3], thus under-enumerating injury-related mortality. By linking mortality and hospital separation data collections a longer follow-up period of mortality post-discharge can be examined to try and account for all mortality attributable to injury. This also has implications for trauma support service delivery and service planning, including injury compensation.

Many factors have been associated with higher injury mortality rates, including injury type [4, 5], increasing injury severity [5], older age [5, 6], comorbid conditions [4, 7] and treatment at hospitals without Level 1 trauma centre facilities [8, 9]. Prior injury and matched non-injured cohort comparison studies have identified that survival is worse and mortality rate ratios are higher for injured compared to non-injured cohorts many months post-injury (i.e. ranging from 1 to 33 years) [10–12], but have not examined cause of death for all types of injuries. This study aims to quantify and describe mortality attributable to injury 12 months after an injury-related hospitalisation using a population-based matched cohort study in Australia.

## Method

This is a population-based matched cohort study of individuals aged ≥18 years using linked emergency department (ED) presentation, hospital admission and mortality records from three Australian states during 1 January 2008 to 31 December 2010. Ethical approval was obtained from each Human Research Ethics Committee associated with each State Health Department. The method for this study has been described elsewhere [13] and an overview is provided here.

### Data sources

The hospitalisation records include information on inpatient admissions from all public and private hospitals in New South Wales (NSW) and Queensland (QLD) and for public hospitals only in South Australia (SA). The hospital admission records contain information on patient demographics, source of referral, diagnoses, external cause(s), hospital separation type, and clinical procedures. Diagnoses and external cause codes were classified using the International Classification of Diseases, 10th Revision, Australian Modification (ICD-10-AM) [14].

The ED presentation records contain information collected from public hospital EDs in NSW, QLD, and SA. Data collected included patient demographics, arrival and departure dates, triage category, type of visit and clinical procedures. Mortality data were obtained from the Registry of Births, Deaths and Marriages in the three Australian states and was used to identify date and cause of death. Cause of death information was not available for *n* = 40 (0.3%) individuals (*n* = 33 injured and *n* = 7 non-injured) in NSW.

### Injured population

The injured population included all people in the hospital admission records in 2009 with a principal diagnosis of injury (ICD-10-AM: S00-T75 or T79). If these people had more than oneinjury-related hospital admission in that year, the first injury was identified as the index admission.

### Non-injured comparison population

A comparison cohort of people aged ≥18 years who were not hospitalised for an injury during 2009 was randomly selected from electoral rolls covering the study region. Because it is compulsory to vote in Australia, most individuals aged ≥18 years are registered on the electoral roll and thus the comparison group was population based. Individuals in the non-injured cohort were selected by each state data linkage centre, with 1:1 matching performed for the date of the index injury admission of their injured counterpart on age, gender, and postcode of residence. All ED, hospital admission and mortality records for the non-injured cohort were identified.

### Data linkage

Data custodians in each state identified the index injury-related hospitalisations in 2009. State-based data linkage centres probabilistically linked all ED, hospitalisation and mortality records of the injured cohort residing in their respective states. Each state data linkage centre was provided an extract of the electoral roll to randomly select the matched, non-injured comparison group. All records from NSW and SA were provided to the Centre for Data Linkage (CDL) and records for these two states were probabilistically linked to identify any cross-border health care use by either injury cases or their matched counterparts. The data linkage centres used identifying information (e.g. name, address, date of birth, gender) to create a unique identifier for each person identified in the linkage process.

### Identification of comorbidities

A 12-month look back period from the admission date of the index injury admission was used for the identification of comorbidities for both the injury cases and their matched counterparts. Relevant comorbidities for all study participants were identified using diagnosis classifications from the hospitalisation records [15] and coded according to the The Charlson Comorbidity Index (CCI). The CCI was treated as a categorical variable and categorised as severe comorbidity (CCI ≥ 3), mild comorbidity (CCI = 1 or 2) and no reported comorbidity (CCI = 0). Specific health conditions associated with injury risk and poor recovery [16, 17], including mental health conditions (ICD-10-AM: F20-F50), alcohol misuse and dependence (ICD-10-AM: F10, Y90, Y91, Z50.2, Z71.4, Z72.1) and drug-related dependence (ICD-10-AM: F11-F16, F19, Z50.3, Z71.5, Z72.2) were also identified using hospitalisation records.

### Identification of urban and rural location of residence

The Australian Statistical Geographical Standard Remoteness Area was used to identify rural and urban residents. It assigns residents to one of five categories (i.e. major cities, inner regional, outer regional, remote and very remote) using defined index scores of distance to service centres of various sizes [18]. The score is initially calculated on a 1 kilometre grid, and then the mean value for each Census Collection District is aggregated to form the remoteness areas. The five categories were collapsed into two categories: urban (i.e. major cities) and rural (i.e. inner regional, outer regional, remote, and very remote).

### Pre-existing health service use and injury type identification

The number of ED presentations, the number of hospital admissions and total length of stay (LOS) in hospital 1 year preceding the index injury-related admission were identified for both the injured cohort and their non-injured counterparts. Specific types of injury were identified using the principal injury diagnosis, including traumatic brain injury (TBI) (ICD-10-AM: S06); hip fractures (ICD-10-AM: S72.0-S72.2); and the relevant ICD-10-AM classifications for nature of injury including all fractures, open wounds, injury to internal organs, superficial injuries, poisoning by drugs, medicaments and biological substances, burns, injury to nerves and spinal cord, and all other injury types.

### Injury severity and mortality

Injury severity was estimated using the International Classification of Disease Injury Severity Score (ICISS) by applying previously developed survival risk ratios (SRR) to each individual’s injury diagnosis classifications [19]. The ICISS is derived for each person by multiplying the probability of survival for each injury diagnosis using SRRs calculated for each injury diagnosis. [19] Three severity levels were used to define minor (≥0.99), moderate (0.941–0.99) and serious (≤0.941) injury [20].

Twelve-month mortality was calculated from the admission date of the index injury admission for both the injury cohort and their matched non-injured counterparts. All individuals were followed either to their death or to the end date of the study timeframe (up to 365 days following the index injury admission). Cause of mortality was identified using the underlying cause and up to 20 antecedent cause of death fields and categorised into eight sub-groups of cardiovascular disease (ICD-10: I00-I52, I70-I99), cerebrovascular disease (ICD-10: I60-I69), respiratory infections (ICD-10: J00-J22), non-respiratory infections (ICD-10: A00-B99, N39.0), malignant neoplasms (ICD-10: C00-C96), non-infectious respiratory diseases (ICD-10: J30-J99), injuries (ICD-10: S00-T75, T79 and/or V01-Y98), and all other causes of death.

#### Data management and analysis

All analyses were performed using SAS version 9.4 [21]. All hospital episodes of care related to the one injury hospitalisation (or other hospitalisation for non-injured cohort) were linked to form a period of care (i.e. all episodes of care related to the original (or index) injury until discharge from the health system). The study cohort contained 167,600 injured individuals with matched non-injured individuals. For 1011 deaths the date of death was logically implausible, and both these individuals and their matched pair were excluded from this analysis.

A Kaplan-Meier plot of survival estimates and a log rank test was used to compare survival distributions of the injured individuals and their matched non-injured counterpart. Cox proportional hazard regression was used to examine the effect of risk factors on survival. The estimated hazard ratios from the Cox proportional hazard model were used as estimates of mortality rate ratios. Non-proportionality was examined using plots of the negative log of the estimated survivor function against time and the log of the negative log of the estimated survivor function against log time [22].

The number of ED attendances, the number of hospital admissions and the pre-injury cumulative hospital LOS in the 12 months preceding the index injury were correlated and thus only the log of pre-injury cumulative hospital LOS was used. Variables included in the final model were age group, sex, urban status, number of Charlson comorbidities (i.e. 0, 1–2 or ≥3), alcohol use and dependence, mental health conditions, drug-related dependence and the log of pre-injury cumulative hospital LOS. These were examined by injury type and injury severity. Matching variables were included in the model to control for any possible confounding by the matching variables [23]. Hazard ratios (HR) and 95% confidence intervals (95%CI) were calculated. The attributable risk percent (AR%) was calculated by subtracting 1 from the adjusted rate ratio, divided by the adjusted rate ratio, multiplied by 100 [24].

## Results

There were 166,589 individuals who were injured in 2009 and admitted to hospital in NSW, SA or QLD with a matched non-injured comparison. Males represented 57.0% of those injured, 29.8% were aged 18–34 years, 38.0% were aged 35–64 years, 32.1% were aged ≥65 years, and 65.0% resided in an urban location. The mean age for the injured and non-injured comparison cohorts was 51.8 years (SD = 23.2). The injured individuals had poorer pre-injury health in the year preceding the index injury than the non-injured group. The injured individuals had a higher number of ED attendances, hospital admissions and pre-injury cumulative hospital LOS in the 12 months preceding the index injury than their non-injured counterparts. The injured individuals also had significantly higher proportions of Charlson comorbid conditions, mental health diagnoses, and alcohol and drug-related dependence than the non-injured group (Table 1).

**Table 1:** Demographic and health service use characteristics of injury-related hospitalisations of individuals aged 18+ years and matched non-injured comparison cohort in 2009, co-morbidity and mortality data in Australia

Characteristic	Injury cohort (*n* = 166,589)		Non-injured comparison cohort (*n* = 166,589)		
	n	%	n	%	
Australian state					
New South Wales	90,833	54.5	90,833	54.5	
Queensland	58,833	35.3	58,833	35.3	
South Australia^a^	16,923	10.2	16,923	10.2	
Gender					
Male	94,939	57.0	94,939	57.0	
Female	71,650	43.0	71,650	43.0	
Age group					
18–24	23,350	14.0	23,350	14.0	
25–34	26,395	15.8	26,395	15.8	
35–44	24,168	14.5	24,168	14.5	
45–54	21,452	12.9	21,452	12.9	
55–64	17,642	10.6	17,642	10.6	
65–74	14,322	8.6	14,322	8.6	
75–84	19,862	11.9	19,862	11.9	
85+	19,398	11.6	19,398	11.6	
Location of residence					
Urban	108,309	65.0	108,309	65.0	
Rural	58,280	35.0	58,280	35.0	
Pre-existing health service use					
Number of emergency department presentations in the 12 months prior to the index injury date	152,183	-	40,469	-	-
Number of hospital admissions in 12 months prior to the index injury date	153,200	-	66,766	-	-
Total hospital length of stay prior to the index injury date (days)	759,287	-	277,099	-	-
Charlson comorbidity conditions					*χ* ^2^ (df)
0	140,748	84.5	157,930	94.8	10004.4 (2)*
1–2	21,567	13.0	8145	4.9	
≥3	4274	2.6	514	0.3	
Other health conditions					
Mental health diagnoses^b^	10,390	6.2	1498	0.9	6897.1 (1)*
Alcohol misuse and dependence	12,560	7.5	727	0.4	10975.8 (1)*
Drug-related dependence	3165	1.9	369	0.2	2235.8 (1)*

*p < 0.0001

^a^ Includes people hospitalised in public hospitals in South Australia only. ^b^ Includes depression, schizophrenia, bipolar and anxiety disorders

**Fig. 1:**
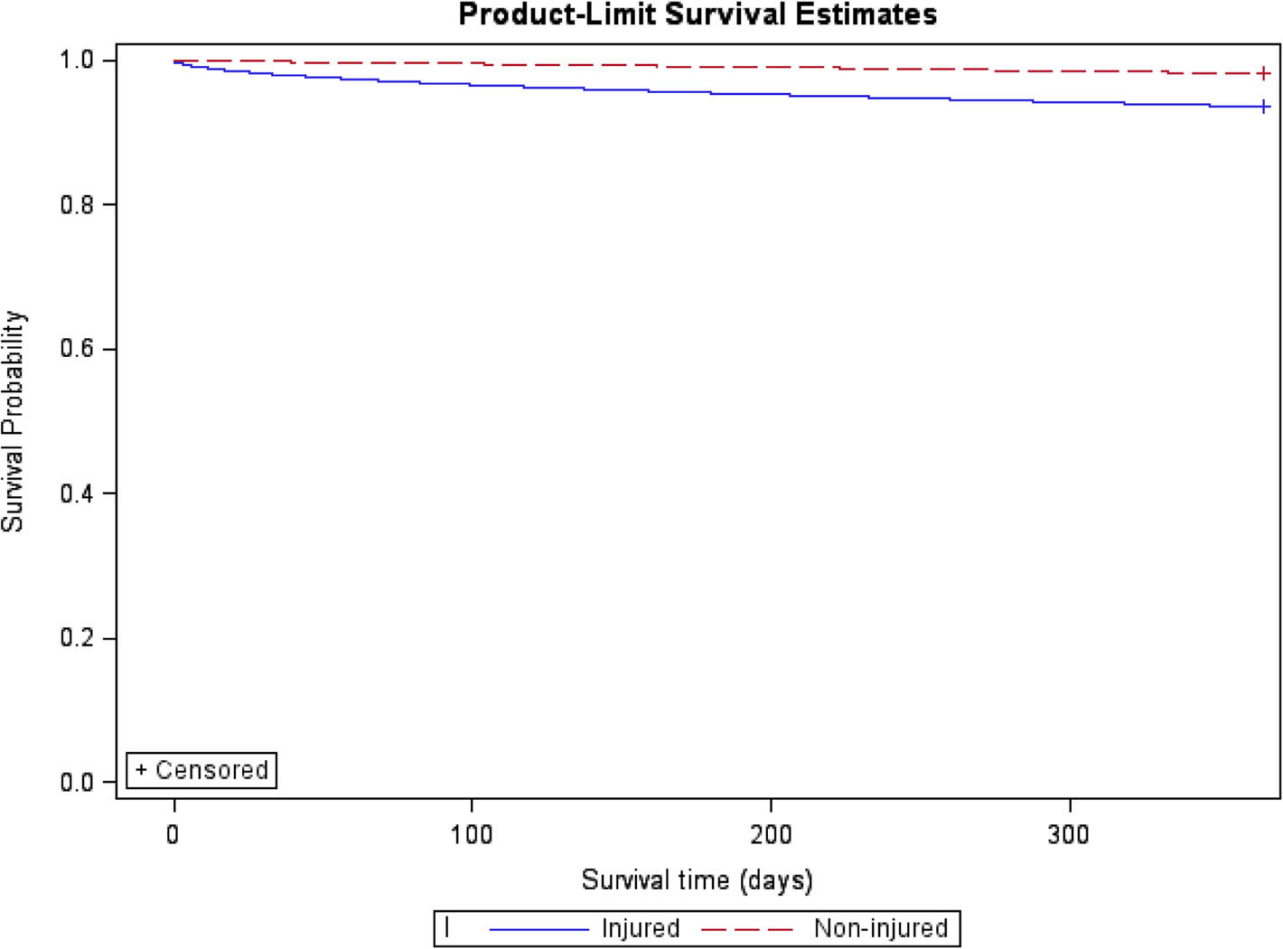
Survival of hospitalised injured individuals aged 18+ years and matched non-injured comparison cohort within the 12 months post the index injury date, linked hospitalisation and mortality data in Australia

The injured individuals experienced higher crude and adjusted mortality rate ratios than the non-injured comparison population at the end of the 12 months following the index injury. Adjusting for pre-injury health status and pre-injury cumulative hospital LOS reduced the mortality risk. Ninety-two percent of mortality within 30-days of hospital admission and 66% of mortality at 12 months was likely to be attributable to injury. Individuals with a traumatic brain injury (MRR 7.58; 95% CI: 5.92–9.70) or injury to internal organs (MRR 7.38; 95% CI: 5.90–9.22) were 7 times more likely to die than the non-injured group, with 87% and 86% of mortality at 12 months likely to be attributable to these injuries, respectively. Poisoning (83%) and burns (75%) also had a high proportion of deaths at 12 months likely to be attributable to the original injury. Individuals with a hip fracture had 3.7 times the adjusted mortality rate ratio than the non-injured group and 73% of mortality at 12 months was likely to be attributable to the hip fracture. The attributable risk of mortality increased with increasing injury severity and decreased with increasing age (Table 2). The log rank test showed a significant difference in survival between injured and non-injured individuals (*χ* ^2^ = 4545.5, df = 1; *p* < 0.0001), with injured individuals experiencing worse survival at 12 months post the index injury admission date (Fig. 1).

**Table 2:** Mortality rate ratios by injury type and severity for injury-related hospitalisations of individuals aged 18+ years and matched non-injured comparison cohort within the 12 months post the index injury date, linked hospitalisation and mortality data in Australia

	Injured No. deaths	Non-injured No. deaths	Unadjusted mortality rate ratio	95%CI	Adjusted mortality rate ratio^a,b^	95%CI	Adjusted attributable risk%^a,b^
Mortality							
Within 30 days	3015	205	15.07*	13.06–17.39	12.13*	10.29–14.29	92
Within 3 months	5327	659	8.69*	7.99–9.45	6.31*	5.75–6.93	84
Within 6 months	7471	1368	6.00*	5.65–6.37	4.27*	4.00–4.57	77
Within 12 months	10,638	2970	4.07*	3.89–4.24	2.90*	2.76–3.04	66
Injury type							
Fracture	5005	1504	3.90*	3.67–4.15	2.80*	2.61–3.01	64
Hip fracture	2390	581	5.34*	4.82–5.91	3.67*	3.27–4.12	73
Open wound	1312	417	3.53*	3.14–3.97	2.57*	2.25–2.93	61
Injury to internal organs	991	141	9.48*	7.74–11.60	7.38*	5.90–9.22	86
Traumatic brain injury	870	119	10.22*	8.18–12.77	7.58*	5.92–9.70	87
Superficial injuries	797	267	3.27*	2.83–3.78	2.21*	1.87–2.60	55
Poisoning by drugs, medicaments and biological substances	479	50	10.56*	7.78–14.34	6.06*	3.93–9.33	83
Burns	69	12	5.75*	3.12–10.61	4.06*	1.71–9.63	75
Injury to nerves and spinal cord	32	15	2.07**	1.12–3.83	1.93	0.88–4.24	48
All other and unspecified injuries	1953	564	3.99*	3.61–4.41	2.76*	2.47–3.08	64
Injury severity score (ICISS range)							
Minor (≥0.99)	1427	597	2.57*	2.33–2.84	1.82*	1.62–2.05	45
Moderate (0.942- < 0.99)	4179	1277	3.68*	3.44–3.93	2.54*	2.35–2.73	61
Serious (<0.942)	5032	1096	5.85*	5.43–6.29	4.17*	3.85–4.52	76
Age group							
18–24	94	7	13.43*	6.23–28.93	28.08*	7.66–103.00	96
25–34	168	8	21.00*	10.33–46.68	25.59*	8.33–78.63	96
35–44	245	23	10.65*	6.95–16.33	7.18*	4.06–12.71	86
45–54	333	56	6.07*	4.56–8.07	4.63*	3.11–6.88	78
55–64	473	69	6.87*	5.33–8.84	5.00*	3.45–7.25	80
65–74	989	186	5.56*	4.74–6.53	2.89*	2.36–3.53	65
75–84	3056	727	4.84*	4.44–5.28	3.16*	2.87–3.49	68
85+	5280	1894	3.35*	3.16–3.54	2.58*	2.42–2.75	61

*p < 0.0001; **p < 0.02

^a^Adjusted for age group, sex, urban status, number of Charlson comorbidities (i.e. 0, 1–2 or ≥3), alcohol use and dependence, mental health conditions, drug-related dependence and the log of pre-injury cumulative hospital LOS. ^b^Age group analyses adjusted for sex, urban status, number of Charlson comorbidities (i.e. 0, 1–2 or ≥3), alcohol use and dependence, mental health conditions, drug-related dependence and the log of pre-injury cumulative hospital LOS

The attributable risk percentages ranged from 94% for malignant neoplasms to 99% for injuries where the cause of death was likely to be attributable back to the original injury (Table 3). Of the injured individuals aged ≥ 75 years with a cause of death of respiratory infection (*n* = 1309), their most common principal injury diagnoses were hip fracture (*n* = 388; 29.6%), open wounds (*n* = 168; 12.8%), superficial injury (*n* = 108; 8.3%), injury to internal organs (*n* = 75; 5.7%), and TBI (*n* = 67; 5.1%). Hip fractures were also a common principal diagnosis of injury for injured individuals ≥ 75 years with a cause of death of non-respiratory infections, such as sepsis (*n* = 237; 26.9% with 882 total deaths) and non-respiratory diseases, such as pneumothorax (*n* = 563; 26.9%, with 2096 total deaths).

**Table 3:** Mortality rate ratios by cause of death for injury-related hospitalisations of individuals aged 18+ years and matched non-injured comparison cohort within the 12 months post the index injury date, linked hospitalisation and mortality data in Australia

Cause of death^a^	Injured No. deaths	Non-injured No. deaths	Unadjusted mortality rate ratio	95%CI	Adjusted mortality rate ratio^b^	95%CI	Adjusted attributable risk%^b^
Underlying and/or antecedent cause of death^c^							
Injuries	2548	166	93.69*	64.12–136.89	81.06*	51.87–126.67	99
Cardiovascular disease	5926	537	25.62*	22.43–29.26	18.35*	15.88–21.22	95
Cerebrovascular disease	1887	159	26.70*	21.00–33.95	22.51*	16.87–30.05	96
Respiratory infections	1487	133	27.98*	21.22–36.89	21.04*	15.48–28.60	95
Non-respiratory infections	1079	87	30.20*	21.57–42.29	20.43*	14.22–29.38	95
Malignant neoplasms	2082	161	26.91*	21.41–33.84	16.16*	12.63–20.68	94
Non-infectious respiratory diseases	2535	204	30.25*	24.28–37.69	19.49*	15.42–24.65	95
All other deaths	735	43	40.16*	25.16–64.11	50.68*	24.77–103.72	98

*p < 0.0001

^a^Cause of death was not able to be provided for *n* = 40 individuals in NSW. ^b^Adjusted for age group, sex, urban status, number of Charlson comorbidities (i.e. 0, 1–2 or ≥3), alcohol use and dependence, mental health conditions, drug-related dependence and the log of pre-injury cumulative hospital LOS. ^c^Cause of death was not mutually exclusive

## Discussion

Injury mortality remains a substantial burden, with GBD estimates indicating that injury was accountable for approximately 4.8 million deaths worldwide in 2013 [25]. This study identified that injured individuals had worse survival at 12 months after their injury admission compared to a matched non-injured group and that higher mortality rates largely remained for injured individuals after adjusting for pre-injury health conditions. Reasons why the risk of mortality remains elevated for the injury cohort is unclear, but suggests that there are likely to be long-term chronic consequences of sustaining a traumatic injury [26].

Injured individuals had the highest elevated mortality risk within 30 days of their index injury admission (MRR 12.13; 95% CI: 10.29–14.29), but at 12 months following their injury admission they still had twice the risk of mortality compared to the non-injured group. Attributing a death to injury-related causes soon after the injury event occurred is likely to be relatively straightforward. However, for some individuals, such as those who also have chronic health conditions or older people who might be less likely to recover quickly from injury compared to younger individuals [27, 28], they can have an elevated risk of mortality following their injury for a considerable period of time [3, 11, 28]. In the current study, nearly all mortality within 30 days of the index injury admission was likely to be attributed to the initial injury, with 66% of mortality at 12 months still likely to be attributable to the original injury. This suggests that at up to 1 year following a hospitalised injury, individuals can still have an elevated mortality risk.

Those individuals who sustained minor, moderate and severe injuries had an elevated risk of mortality at 12 months after the index injury admission compared to the non-injured group. Previous studies of mortality at 10 years following an injury have also shown that severe injuries have a higher attributable mortality risk [10], while a study of 10 year mortality following a burn injury did not identify a positive relationship between burn severity and mortality [12]. Duke and colleagues [12] proposed that this might be due to individuals with the most severe burns dying during their initial hospitalisation and/or that the severely injured group of individuals could be a more robust population.

Individuals who sustained a TBI had a 7 times elevated risk of mortality compared to the non-injured group at 12 months after the injury. Cameron et al [10] found, in a study of all-cause mortality after hospitalised injury with a matched non-injured comparison group, that individuals who sustained a brain injury had 3 times elevated risk of mortality compared to the non-injured group even up to 10 years post the initial injury. Likewise, Baguley et al [29], in a comparison of survival of TBI patients with the general population, identified that TBI patients had a 5-fold increased risk of death and McMillian et al [30], in a matched case-control study of injured patients with and without head injury, found an elevated risk of mortality for patients with head injury up to 13 years post-injury.

The original injury event was identified as likely to be a contributory cause for up to 99% of mortality within 12 months of the index injury hospitalisation. There was an elevated risk for all specific causes of death following an injury. That 95% of deaths due to respiratory infections were likely to be attributable back to the injury event is not surprising. Older individuals, in particular, who died sometime after their injury, often have their deaths attributed to other underlying conditions sustained during their recovery period, such as pneumonia [1, 2], which ultimately under-enumerates mortality related to injury.

The current study used underlying and antecedent causes of death to identify cause of death. While this resulted in cause of death that was not mutually exclusive, only using the underlying cause would have underestimated injury as a potential contributory cause of death. Previous research has identified that only using an underlying cause of death under-enumerates injury-related deaths [31], with one study finding that, of injured individuals who died in hospital, only 48% of deaths had an underlying injury-related cause of death classification in their death certificate [32]. There is a need to take into account delayed mortality likely to be related to the initial injury when estimating the extent of injury-related mortality post-hospitalisation [10]. This has implications for the length of trauma patient follow-up studies, for information provided to individuals and their families following trauma treatment [28], for the support services provided to trauma patients post-discharge [12], for injury compensation, and for estimating the DALY in terms of calculating the YLL. Sensitivity estimates of the effect on the DALY of varying YLL could be examined using the injury burden calculator [33].

There were several limitations associated with the current study. It was possible to undertake cross-border record linkage in only two states, which may have resulted in some individuals residing near state borders using health services that may have changed the case-comparison group status of an individual or resulted in additional health service use being recorded for an individual. No private hospital injury hospitalisation data was obtained from SA which will under-enumerate the number of injury hospitalisations, neither was private hospital ED presentation data able to be obtained. However, 81% of hospital separations for injury occur at public hospitals in Australia [34]. It is possible that equity of access to health services and hospital admission policies played a role in whether an individual presented and/or was admitted to hospital [35] and this would have had an impact on injury and comparison cohort selection. There were wide confidence intervals for the adjusted mortality rate ratios for individuals aged less than 34 years and these results should be interpreted with caution.

It is compulsory to vote in Australia, so electoral rolls serve as good population lists for population based sampling for people 18 years or older. However, there are some individuals who do not enrol to vote (e.ge. young people who have not yet enrolled and older people who may be incapacitated [36]) and this may have restricted the selection of the comparison cohort. The number of comorbidities was likely to be under-enumerated from the hospitalisation data as only comorbidities that were relevant to the present hospital episode of care are usually reported. However, in using a 1 year look-back period to identify comorbid conditions, it is likely that better prevalence estimates of comorbid conditions were able to be generated [37]. Inconsistency and/or misclassification in hospitalisation records is likely to exist, but data validity was not able to be assessed. There is also likely to be some degree of error in the record linkage process.

## Conclusion

Individuals who are hospitalised after sustaining an injury can have up to twice the mortality risk 12 months following their injury, suggesting that there are chronic consequences of traumatic injury. Longer follow-up after injury hospitalisation is needed to consider deaths likely to be attributable to the injury that occur post-discharge. Better enumeration of long-term injury-related mortality will improve YLL estimates that then have the potential to improve estimates of the injury burden.

## Abbreviations

CCI: Charlson Comorbidity Index
DALY: Disability Adjusted Life Year
ED: Emergency Department
GBD: Global Burden of Disease
ICD-10-AM: International Classification of Diseases, 10th Revision, Australian Modification
ICISS: International Classification of Disease Injury Severity Score
LOS: Length of stay
MRR: Adjusted mortality rate ratios
NSW: New South Wales
QLD: Queensland
SA: South Australia
TBI: Traumatic Brain Injury
YLD: Years of Life Lost to Disability
YLL: Years of Life Lost
